# Hook-shaped distal common bile duct due to peptic ulcer mimicking cholangiocarcinoma

**DOI:** 10.1016/j.ijscr.2024.110456

**Published:** 2024-10-13

**Authors:** Sebei Amine, Ouadi Yacine, Atri Souhaib, Ben Mahmoud Ahmed, Haddad Anis, Montasser Kacem

**Affiliations:** Department of Surgery A La Rabta Hospital, Tunis, Tunisia; Faculty of Medicine of Tunis, Tunis El Manar University, Tunis, Tunisia

**Keywords:** Peptic ulcer, Duodenal stenosis, Cholangiocarcinoma, Bilio-digestive fistula, Choledoco-duodenal fistula

## Abstract

**Introduction and importance:**

Internal and spontaneous bilio-digestive fistulas, without primary biliary disease, are an infrequent complication of the upper digestive tract. We report a case of a Hook-shaped distal common bile duct due to peptic ulcer mimicking cholangiocarcinoma.

**Case report:**

A 63-year-old patient, with no medical history, presented to the emergency department with recent jaundice. Laboratory tests showed cholestasis, and high bilirubin levels. Cholangio-MRI found a stricture in the distal common bile duct with multiple hepatic lesions which could be abscesses or secondary lesions. An endoscopic ultrasound was attempted to confirm the malignant nature of the CBD stricture but it was not possible due to a duodenal stenosis. Peptic ulcer stenosis complicated with distal CBD retraction and choledochoduodenal fistula was then suspected. The patient was put on proton pump inhibitors and antibiotics with favorable clinical, biological and endoscopic evolution.

**Discussion:**

Choledoco-duodenal fistulas resulting from peptic ulcers are rare.

The incidence of fistulas associated with peptic ulcer disease has declined significantly in recent years. This reduction is largely attributed to advancements in medical treatment for ulcers. Patients with choledoco-duodenal fistula often present with nonspecific symptoms, making the condition challenging to diagnose.

**Conclusion:**

Spontaneous bilio-digestive fistulas, in the absence of primary biliary disease, are a rare complication of the upper digestive tract. Conservative treatment is recommended for CDF caused by duodenal peptic ulcers.

## Highlights

• Spontaneous bilio-digestive fistulas, in the absence of primary biliary disease, are a rare complication of the upper digestive tract.
• Choledoco-duodenal fistulas caused by peptic duodenal ulcers are becoming increasingly uncommon.
• The prognosis for patients treated medically is good, as complications such as angiocholitis and biliary sequelae are rare and do not justify prophylactic surgical treatment.
• Conservative treatment is recommended for choledoco-duodenal fistulas caused by duodenal peptic ulcers.

## 1 Introduction and importance

Internal and spontaneous bilio-digestive fistulas, without primary biliary disease, are an infrequent complication of the upper digestive tract. These fistulas are predominantly cholecystoduodenal, mainly resulting from cholelithiasis, and account for 90 % of all bilio-digestive fistulas [1]. Exceptionally, choledoco-duodenal fistulas can be caused by duodenal peptic ulcers. Therefore, due to the rarity of this complication nowadays, we present the case of a hook shaped distal common bile duct (CBD) caused by a duodenal peptic ulcer and mimicking a cholangiocarcinoma in order to consider this differential diagnosis when there is a case of jaundice with history of peptic ulcer. This case was reported in line with the SCARE criteria [2].

## 2 Case report

**Fig. 1:**
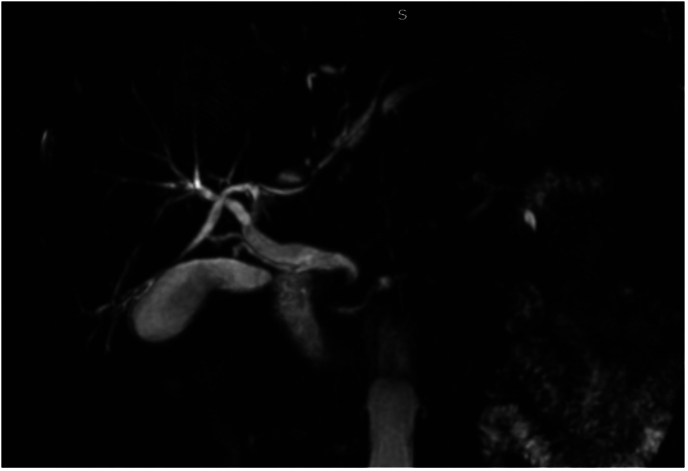
MRI imaging showing the hook-shaped distal CBD.

A 63-year-old patient, with no medical history, presented to the emergency department with recent jaundice, and vomiting. Physical examination showed fever and tenderness in the right hypochondrium with no palpable mass. Laboratory tests showed a CRP at 78 mg/l associated with conjugated hyperbilirubinemia at 40 umol/l. Alkaline phosphatase was elevated at 808 UI/l and YGT was elevated at 449 UI/l. Abdominal ultrasound revealed a dilated CBD at 12 mm, with visibility of intrahepatic ducts. Cholangio-MRI found a stricture in the distal common bile duct with multiple hepatic lesions which could be abscesses or secondary lesions (Fig. 1).

**Fig. 2:**
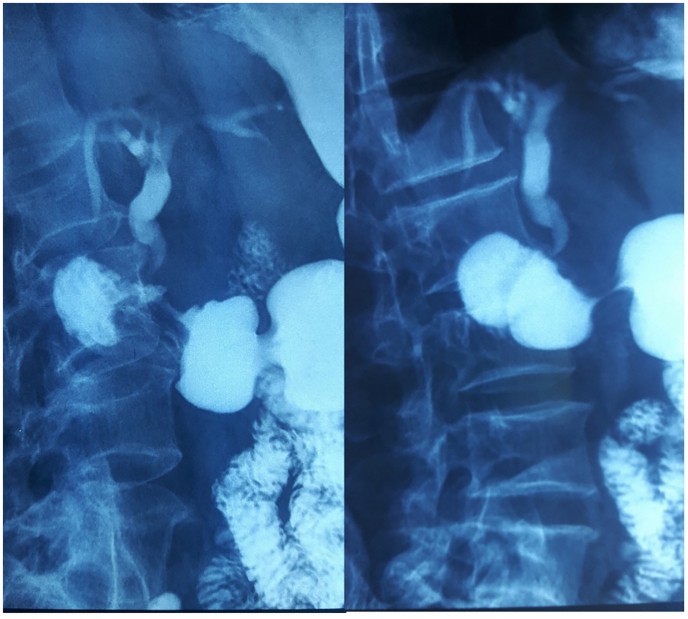
Barium series showing bile ducts indicating choledochoduodenal fistula.

The diagnosis of distal CBD cholangiocarcinoma was suspected and tumor markers (Ca 19-9 and ACE) were done but were negative. An echo endoscopy was attempted to confirm the malignant nature of the CBD stricture but it was not possible due to a duodenal stenosis. The patient was reinterrogated and revealed a history of ulcer-like abdominal pain that has been ongoing for ten years. Duodenal biopsy was negative for malignant cells but positive for *H. pylori* bacteria. We reviewed the Cholangio-MRI and we noticed that the CBD had a hook shaped distal portion. Peptic ulcer stenosis complicated with distal CBD retraction and choledochoduodenal fistula was then suspected. Barium study showed normal passage in the small bowel but an unusual passage in the biliary ducts revealing a hook-shaped distal CBD (Fig. 2). The patient was put on proton pump inhibitors and antibiotics with favorable evolution: regression of jaundice and resolution of the abdominal pain and vomiting.

After 3 months of proton pump inhibitors treatment, follow-up upper GI endoscopy showed normal passage into the duodenum, with echo endoscopy revealing no abnormal thickening or stenosis in the distal CBD. Stool test for *H. pylori* was done to confirm the eradication of the bacteria.

## 3 Discussion

Choledoco-duodenal fistula, a subtype of choledocoenteric fistula, occurs when an abnormal connection forms between the common bile duct and the duodenum [1]. The primary cause of this condition is inflammation in the bile duct, typically due to gallstones. These stones can erode through the bile duct wall, leading to the formation of a fistula [3]. Less commonly, choledoco-duodenal fistulas can result from other factors, such as malignancies (tumors in the bile duct or pancreas), peptic ulcers, or inflammatory conditions affecting nearby organs, like Crohn's disease [4].

The incidence of fistulas associated with peptic ulcer disease has declined significantly in recent years. This reduction is largely attributed to advancements in medical treatment for ulcers, including the widespread use of proton pump inhibitors and antibiotics to eradicate *Helicobacter pylori*, a common cause of peptic ulcers [5]. These medications have greatly reduced the occurrence of severe ulcerations that can lead to fistula formation.

Patients with choledoco-duodenal fistula often present with nonspecific symptoms, making the condition challenging to diagnose. Common symptoms include recurrent episodes of cholangitis (inflammation of the bile ducts), characterized by fever, abdominal pain, and jaundice (yellowing of the skin and eyes). Some patients may also exhibit abnormal liver function tests, indicating liver involvement. However, many cases are asymptomatic and are discovered incidentally during investigations for other conditions [6].

Diagnostic tools play a crucial role in identifying choledoco-duodenal fistulas. Imaging studies are particularly useful. Pneumobilia, the presence of air within the bile ducts, is a significant radiographic marker found in approximately 50 % of cases. This can be detected on abdominal X-rays or computed tomography (CT) scans [4]. Another useful diagnostic method is the upper gastrointestinal (GI) barium series, where the passage of contrast material into the bile ducts can be observed, confirming the presence of a fistula [1].

The reason why we suspected this complication immediately after the endoscopy is because of the higher rate for peptic ulcer complications in our country. For this complication to occur, the duration of the ulcer disease varies from 7 to 11 years [7]. This was the case for our patient as he had a history of undocumented ulcer-like abdominal pain that has been ongoing for ten years.

The existence of a choledoco-duodenal fistula does not in itself represent a formal indication for surgery. Indeed, given the rarity of severe complications like cholangitis and long-term biliary sequelae, routine preventive surgical intervention for choledoco-duodenal fistulas is not generally recommended. Surgery is usually reserved for cases that do not respond to conservative management [8]. Instead, the primary treatment approach focuses on addressing the underlying conditions that led to the fistula's formation. For instance, in cases where peptic ulcer disease is the cause, treating the ulcer with appropriate medications is the mainstay of therapy. This approach helps reduce inflammation and promotes healing, potentially leading to the closure of the fistula without the need for invasive procedures [5].

## 4 Conclusion

Spontaneous bilio-digestive fistulas, in the absence of primary biliary disease, are a rare complication of the upper digestive tract. Choledoco-duodenal fistulas caused by peptic duodenal ulcers are becoming increasingly uncommon. These fistulas typically present with non-specific symptoms and are usually diagnosed through radiological or endoscopic investigations. The prognosis for patients treated medically is good, as complications such as angiocholitis and biliary sequelae are rare and do not justify prophylactic surgical treatment. Therefore, conservative treatment is recommended for choledoco-duodenal fistulas caused by duodenal peptic ulcers.

## Author contribution

Sebai Amine, CONCEPTUALISATION, REDACTION, DATA CURATION, PROJECT ADMINISTRATION

Ouadi Yacine, CONCEPTUALISATION, REDACTION, DATA CURATION, PROJECT ADMINISTRATION

Atri Souhaib CONCEPTUALISATION, REDACTION,

Ben Mahmoud AhmadREDACTION PHOTOGRAPHY RENDERING, DATA CURATION

Haddad Anis SUPERVISION, VALIDATION, VISUALISATION

Montasser Kacem SUPERVISION, VALIDATION, VISUALISATION

## Consent

Written informed consent was obtained from the patient for publication of this case report and any accompanying images. A copy of the written consent is available for review by the Editor-in-Chief of this journal on request.

## Ethical approval

Not applicable.

## Research registration number

Not applicable.

## Provenance and peer review

Not commissioned, externally pee-reviewed.

## Funding

No sources of funding.

## Conflict of interest statement

All authors declare they have no conflict of interest.
